# RETRACTION: Effects of Phoenix dactylifera against Streptozotocin‐Aluminium Chloride Induced Alzheimer’s Rats and Their In Silico Study

**DOI:** 10.1155/bmri/9797803

**Published:** 2026-07-14

**Authors:** BioMed Research International

RETRACTION: H. Ismail, D. Khalid, S. Bin Ayub, M. U. Ijaz, S. Akram, M.Z. Bhatti, M. M. Taqi, E. Dilshad, S. Anwaar, G. El‐Saber Batiha, S.S. Aggad, F. M. Yousef, and M. De Waard, “Effects of Phoenix dactylifera against Streptozotocin‐Aluminium Chloride Induced Alzheimer’s Rats and Their In Silico Study,” *BioMed Research International*, vol. 2023 (2023). https://doi.org/10.1155/2023/1725638.

The above article, published online on 9 January 2023 in Wiley Online Library (http://wileyonlinelibrary.com), has been retracted by agreement between the journal’s Editor‐in‐Chief, Yoel A. Klug; and John Wiley & Sons Ltd.

The retraction has been agreed following an investigation of the concerns flagged by a third party on PubPeer [1]. The investigation identified concerns related to several values shown in Tables 1, 2, 3, 4, and 5, and the underlying raw data were requested from the authors.

The authors provided the raw data, which were assessed by the publisher and the journal’s editorial board. Following an investigation, it was found that the provided data were not reliable, bringing the validity of the values shown in the abovementioned tables into question.

As a result of the investigation, the data and conclusions of this article are considered unreliable. Therefore, the article must be retracted.

Michel De Waard agrees with this retraction. The other authors disagree with this retraction.
